# Ultra-sensitive and selective fluorescence approach for estimation of elagolix in real human plasma and content uniformity using boron-doped carbon quantum dots

**DOI:** 10.1186/s13065-022-00849-3

**Published:** 2022-08-04

**Authors:** Baher I. Salman, Ahmed I. Hassan, Yasser F. Hassan, Roshdy E. Saraya

**Affiliations:** 1 Pharmaceutical Analytical Chemistry Department, Faculty of Pharmacy, Al-Azhar University, Assiut Branch, Assiut, 71524 Egypt; 2 Pharmaceutical Analytical Chemistry Department, Faculty of Pharmacy, Port Said University, Port Said, 42511 Egypt

**Keywords:** Elagolix, Spectrofluorimetric, B@CQDs, Human plasma, Content uniformity test

## Abstract

Elagolix (ELX) is an orally administered non-peptidic GnRH antagonist that has been approved by the Food and Drug Administration in 2018 for the treatment of endometriosis pain. A sensitive and selective method for estimating elagolix (ELX) in human plasma and content uniformity was developed and validated. The spectrofluorimetric technique was used to investigate ELX utilizing boron-doped carbon quantum dots (B@CQDs). After gradually adding ELX, the quantum dots fluorescence was enhanced with LOQ of 1.74 ng mL^−1^, the calibration curve between ELX and corresponding fluorescence intensity was found over a range of 4–100 ng mL^−1^. The method was successfully applied in real human plasma with pharmacokinetic study and content uniformity test. The pharmacokinetic parameters as C_max_ were found to be 570 ± 5.32 ng. mL^−1^ after 1 h, t_1/2_ was found to be 6.50 h, and AUC was found to be 1290 ± 30.33 ng. h. mL^−1^. B@CQDs were characterized using variety of instruments. The strategy is simple to implement in clinical labs and therapeutic drug monitoring systems.

**Supplementary Information:**

The online version contains supplementary material available at 10.1186/s13065-022-00849-3.

## Introduction

**Fig. 1:**
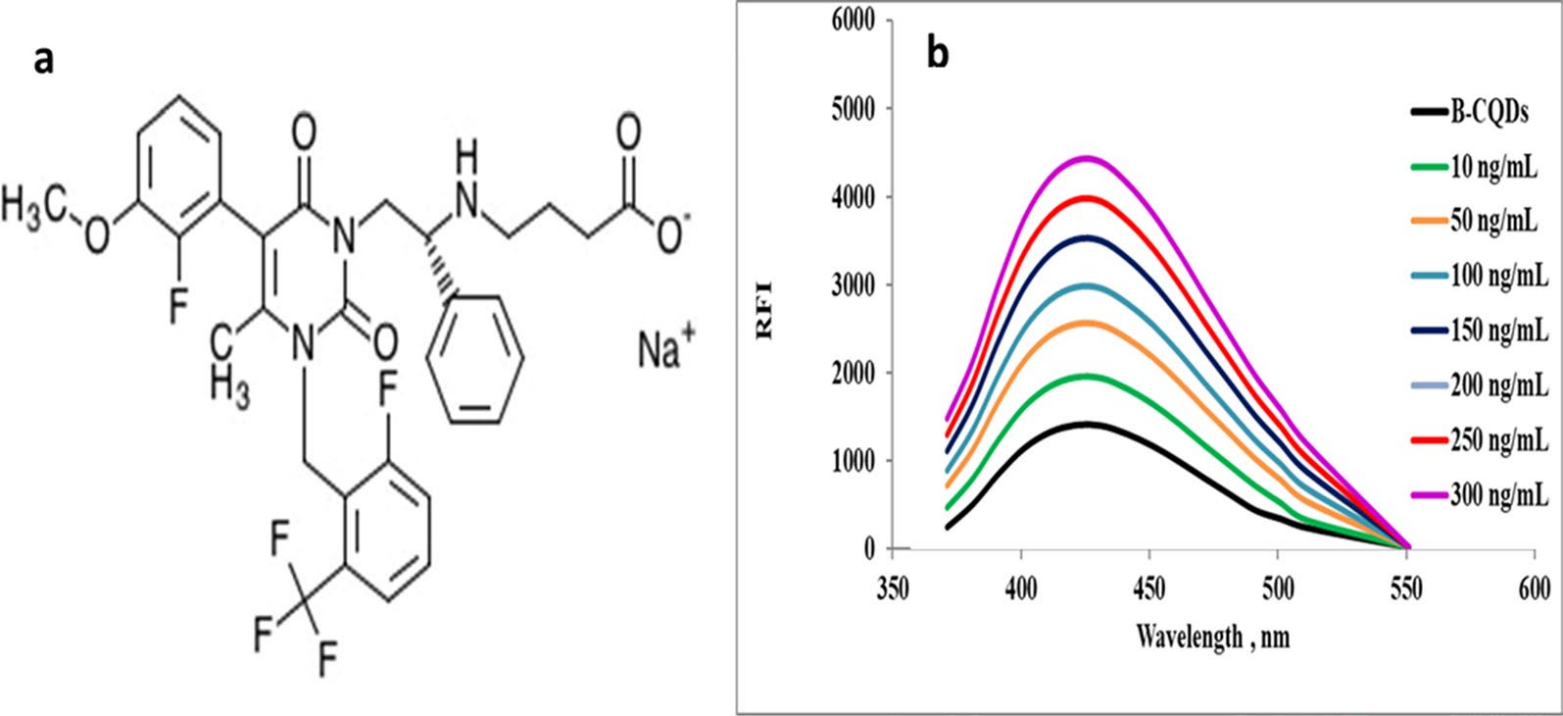
**a** Chemical structure of ELX, **b** emission spectra of reaction B@CQDs with varying concentrations of ELX

ELX (Fig. 1a) is 4-[[(1R)-2-[5-(2-Fluoro-3-methoxyphenyl) -3-[[2-fluoro -6- (trifluoromethyl) phenyl] methyl]-4-methyl-2, 6-dioxopyrimidin-1-yl]-1- phenylethyl]amino] butanoic acid. Endometriosis is the most common gynecological disorder affecting women of reproductive age, except for postmenopausal women [1]. Endometriosis is a condition that develops outside of the uterus because of tissues within it, causing pelvic pain and infertility [2–6]. Pain relievers (nonsteroidal anti-inflammatory drugs) are the first-line treatment for endometriosis pain [7]. ELX is an oral first-generation and short-acting gonadotropin-releasing hormone (GnRH) antagonist drug that was approved by the FDA in 2018 for the treatment of endometriosis pain [8]. ELX is used to relieve pelvic pain by inhibiting GnRH signals by binding competitively to GnRH receptors [8].

Only three methods were reported for the determination of ELX [9–11]. The reported method has many disadvantages as using expensive and sophisticated equipment, expensive solvents, and difficulty of handling. The presented study provides varying advantages other than reported methods as the method is environmentally friendly, time saving, very simple and sensitive with a quantitative range from 4 to 100 ng mL^−1^. Quantum dots is smart materials that have many applications as in industries, science, technology, and pharmaceutical analysis [12–14]. As a result, carbon quantum dots were used to analyze ELX in human plasma (pharmacokinetic assay), content uniformity, and then heteroatoms were introduced into carbon dots synthesis to fine-tune the conduction band position of doped CQDs, which led to the adjustment of the functions used to treat impurities and fluorescence of doped carbon dots. This study describes the practical implementation of a selective and cheap B@CQDs to estimation of ELX in pharmaceutical formulations and human plasma without matrix interference as a proof-of-concept to utilize B@CQDs in clinical laboratories.

### Experimental part

#### Apparatus

the fluorescence was measured using Jasco, FP spectrofluorimetric instrument with a 1 cm quartz cell and slit width 5 nm (USA). The dynamic light scattering measurements (DLS) were scanned by Zetasizer Red badge instrument of ZEN 3600 Nano ZS model (Malvern, UK). Transmission electron microscope (TEM) images were captured on TEM Assembly Parts Power of JEOL JEM-100CX II unit tungsten EM filament 120 (USA). Fourier-transform infrared (FTIR) spectrometer was utilized to determine function groups of B@CQDs (Germany). Ultrasonic Cleaner (USA). Hana pH-meter (China). The powder X-ray diffraction (PXRD) was scanned by Philips X-ray diffractometer. UV–Vis spectra of carbon dots was recorded by Shimadzu 1601PC UV–Vis. The elemental composition of B@CQDs was determined by NEX QC + QuantEZ (50 kV X-ray tube and SDD detector) energy dispersive X-ray spectrometer.

#### Materials and reagents

Elagolix (ELX, 99.99%) was kindly supplied by Hekma pharmaceutical industries, Cairo, Egypt. Orlissa® (200 mg/tablet) was purchased from local pharmacies. FeCl_3_⋅6H_2_O, FeCl_2_⋅4H_2_O, boric acid and glucose, SDS, starch and mannitol were obtained from El-Gomhoria, Egypt. Methanol, ethanol, ethyl acetate and acetonitrile were obtained from El-Nasr Co, Egypt.

A stock solution of ELX was prepared in ultra-pure distilled water by dissolving 10 mg of the drug in a 100-mL volumetric flask to reach a concentration of 100 µg mL^−1^. Stock standard solution was stored for 14 days in the refrigerator at 4 °C. A series of working standard solutions with concentration range between 40 and 1000 ng mL^−1^ were prepared in volumetric flasks using the same solvent.

#### Preparation of human plasma

Human plasma samples were achieved from healthy human volunteers following ethical standards of the responsible committee on human experimentation (institutional and national) and with Helsinki Declaration of 1975, as revised in 2008.

Human plasma was obtained from healthy human volunteers (aged from 25 to 40 years old). Plasma samples was spiked with various concentrations of ELX in centrifugation tubes, 2 mL of acetonitrile [15, 16] was added as protein precipitating agent, and the mixture was centrifuged at 4000 rpm for 15 min. After that, the supernatant completed to 10 mL with ultra-pure water and the procedure was followed.

Six healthy female volunteers were administered Orlissa® tablets (200 mg/tablet) as a single oral dose as part of a pharmacokinetic study. After collecting blood samples at predetermined intervals, the plasma was separated by centrifugation at 4000 rpm for 30 min. The plasma samples were treated as spiked human plasma without the addition of the mentioned drug.

#### Boron doped carbon dots (B@CQDs)

Were synthesized using hydrothermal method [17], 0.3 g boric acid and 0.5 g glucose were dissolved into 50 mL of ultra-pure water via sonication for 20 min. The electric oven was adjusted at 300 ◦C and the mixture was heated for 4 h. After cooling, the resultant mixture was centrifuged at 5000 rpm for 30 min. The material was collected and then dried, 100.0 mg of B@CQDs were transferred into 100.0 mL ultra-pure water. After that, the colloid was sonicated for 1 h and filtered to remove the large particles.

### Procedure for ELX using B@CQDs

Into 5 mL volumetric flasks, 0.5 mL B@CQDs solution, 1.0 mL of B.R. buffer (pH 6.7), and 1 mL of different concentrations of ELX were added. the mixture was diluted to the mark with DDW and mixed. Then, the mixture was measured at 435 nm (excitation at 370 nm) after 10 min.

### Analysis of ELX pharmaceutical dosage form and content uniformity test

Pharmaceutical tablets were prepared as the following procedure, 10 tablets (orlissa®) were weighed and finely powdered. An amount of the powdered tablets equivalent to 10 mg of ELX was transferred into 100-mL volumetric flask followed by addition of 50 mL of ultra-pure water. After that, the solution mixture was sonicated for 5 min followed by filtration and dilution with double distilled water to reach final concentration of 100 µg mL^−1^.

The content uniformity testing was assessed and performed using USP [18, 19]. Each tablet was individually weighed, crushed, and analyzed as the previously mentioned procedure of pharmaceutical tablets.

## Results and discussion

ELX is an orally administered non-peptidic GnRH antagonist used to treat endometriosis pain [8, 9]. The fluorometric technique, as is well known, is a highly selective, quick, and sensitive technique widely employed for pharmaceutical substances [15, 16]. After excitation at 370 nm, the fluorescence of synthesized B@CQDs was seen at 435 nm. After the addition of varying concentrations of ELX to B@CQDs, enhancement of the fluorescence was observed Fig. 1b.

The enhancing reaction mechanism of ELX with B@CQDs, which is based on hydrogen bonding, energy/electron transfer, and electrostatic contact, resulted in an increase in the quantum dots' fluorescence. Because of the hydrogen bonding and electron-donor–acceptor complex between B@CQDs and ELX, as well as the abundance of carboxyl, hydroxyl, and trivalent boron moieties, the B@CQDs' fluorescence was enhanced by combining with ELX. The carboxyl or hydroxyl groups of B@CQDs and the fluorine of ELX form active and strong nearby hydrogen bonds [20, 21].

Furthermore, the electron-accepting representative of trivalent boron stabilized by the carbon skeleton of B@CQDs and the electron-donating character of ELX, which may promote the conjugation of C = C bonds [22], as well as the merging effect of hydrogen bonding and electron-donor–acceptor complex effect, led to an increase in the initiation of massive chromophores and fluorophores [22].

### Characterizations of doped boron carbon quantum dots (B@CQDs)

**Fig. 2:**
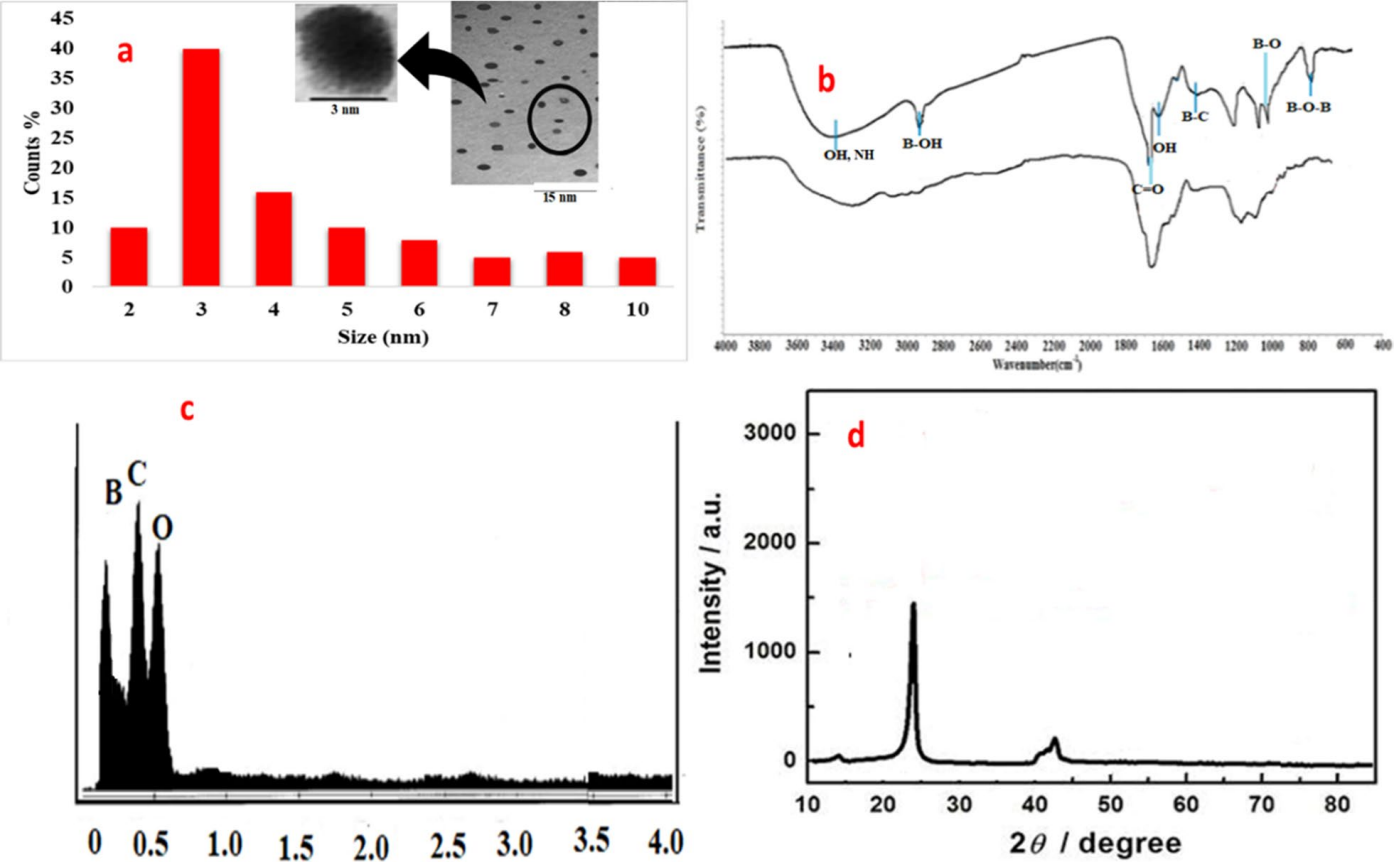
Morphological characters of B@CQDs, **a** TEM image with DLS for B@QDs, **b** FTIR, **c** EDX characterization of B@QDs and **d** PXRD for B@CQDs

Varying morphological characters of the quantum dots were studied, firstly TEM image was carried out to study their particle size. The size of the quantum dots was found to be 3 nm and conformed with DLS spectrum Fig. 2a.

The FTIR for B@CQDs, the bands that emerge at 3412, 1690, 1622, 1095, and 1236 cm^−1^ correspond to (OH), (C = O), (C = C), (C O C), and (CO) respectively [14, 23].

However, B@CQDs FTIR provides characteristic peaks at 2952, 1425, 1031, and 796 cm^−1^ are indicated to (B‒OH), (B‒C), (B‒O), and (B‒O‒B) respectively, which confirms the doping of boron in the carbonaceous structure of the synthesized B@CQDs compared to the undoped CQDs [24] Fig. 2b.

EDX spectrum provides three characteristic peaks for B@CQDs refer to B 20.13% at 0.18 keV, C 49.22% at 0.24 keV and O 30.65% at 0.51 keV as shown in Fig. 2c.

To characterize B@CQDs further, PXRD (Fig. 2d) was employed strong peak at 2θ = 23.9, which corresponds to the graphite phase (002) plane [25] and diffraction peaks at 11.4 (001) and 42.3 (100) for B@CQDs compared with undoped carbon dots Additional file 1: Fig. S1.

**Fig. 3:**
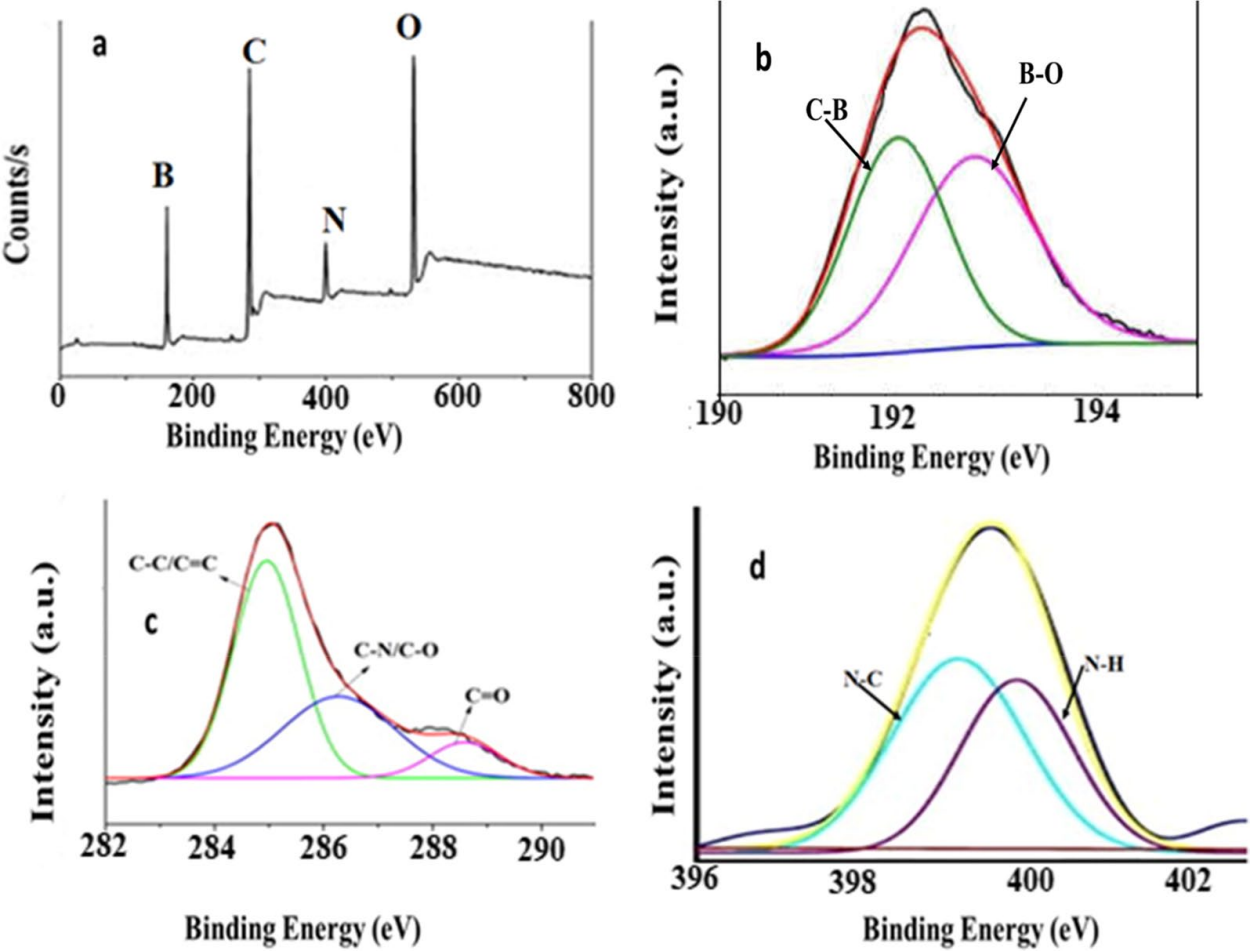
XPS images for B@CQDs, **a** elemental image for B@CQDs, **b** B1s image, c) C 1 s image and d) N 1 s image for B@CQDs

As shown in Fig. 3a, XPS was used for elemental analysis. The B 1 s high-resolution spectra revealed two peaks with binding energies of 192.09and 192.97 eV, which correspond to B–C and B–O, respectively (Fig. 3b). Carboxylic groups are present at 288.20, as well as C–O/C–N and C = C bonds at 285.27 and 284.47, respectively, in the high-resolution C 1 s image (Fig. 3c). The spectrum of N 1 s exhibits two distinct peaks at 399.20 and 400.10 eV, indicating two components for N–C and N–H [26, 27] Fig. 3d.

**Fig. 4:**
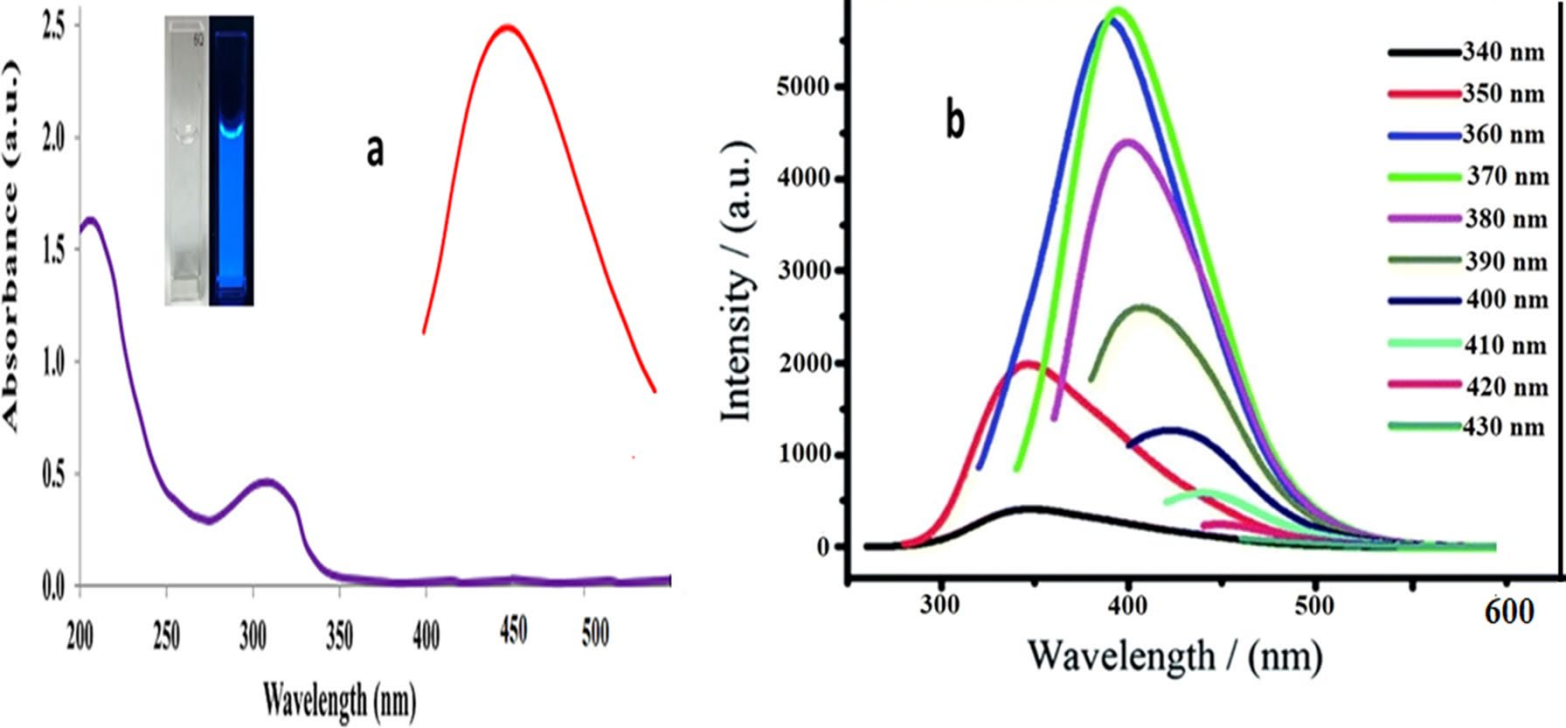
**a** Spectral analysis of B@CQDs, **b** Excitation dependent emission curve

The quantum yield of B@CQDs was determined using the single point method [12, 17]:

$${{\varvec{Q}}}_{{\varvec{X}}}={{\varvec{Q}}}_{{\varvec{s}}{\varvec{t}}}.\boldsymbol{ }\frac{{{\varvec{I}}}_{{\varvec{X}}}}{{{\varvec{I}}}_{{\varvec{s}}{\varvec{t}}}}.\frac{{{\varvec{A}}}_{{\varvec{s}}{\varvec{t}}}}{{{\varvec{A}}}_{{\varvec{X}}}}.\frac{{{\varvec{\eta}}}^{2}}{{{\varvec{\eta}}}^{2}}$$

where, Q_st_ is quantum yield for standard solution (quinine sulphate), (I) is the integrated fluorescence intensity, ${\varvec{\eta}}$ is the refractive index of the water and A is absorption. B@CQDs have quantum yield 38.44%.Spectrophotometric and spectrofluorimetric equipment were used to analyses the quantum dots' spectrum properties. Two peaks at 209 and 312 nm seen in Fig. 4a. These peaks were referred to as the π-π* electronic transition of C = C and the n-π* electronic transition of C = O which are related to the synthesized B@CQDs.Furthermore, B@CQDs provides an emission peak at 435 nm (excitation at 370 nm), which indicates carbon optical properties. Fluorescence (FL) spectra of B@CQ-dots were studied with change wavelength excitation from 340 to 430 nm. Increasing excitation led to a red shift in the emission of B@CQ dots followed by a decrease in RFI, that validates carbon dots excitation-dependent emission [14] Fig. 4b.

Under various settings, the reaction between ELX and B@CQDs was optimized. The influence of pH on fluorescence enhancement was investigated in the range of 5.5 to 7.3, the highest fluorescence intensity was achieved using pH 6.4 ± 0.3 (Additional file 1: Fig. S2a) with a buffer concentration equal to 0.02 M Additional file 1: Fig. S2b.

Furthermore, the effect of B@CQD concentration was investigated from 0.005 to 0.035 mg mL^−1^, the greatest fluorescence obtained using 0.012 mg mL^−1^ and not affected by increasing B@CQD volume. As a result, the optimum concentration of B@CDs was determined to be 0.015 mg mL^−1^ (0.5 mL) Additional file 1: Fig. S2c.

At varied time intervals spanning from 0 to 20 min as in Additional file 1: Fig. S2d, the efficiency of fluorescence amplification in the presence of ELX was studied. The maximum fluorescence increase of B@CQDs was achieved after 8 min, so 10 min was used as the optimum reaction time.

### A suggested reaction mechanism between ELX and B@CQDs

The hydrogen bonding and electron-donor–acceptor complex between B@CQDs and ELX could explain the fluorescence enhancement technique. Based on the two highlighted, active, and strong neighboring hydrogen bonds between the hydroxyl/carboxyl of B@CQDs and fluorine atoms of ELX [14, 17, 21], the existence of carboxyl, hydroxyl, and trivalent boron groups offers viability to conjugate with the referenced analyte. Strong intermolecular hydrogen bonds can form a center that connects two molecules that are next to each other. The types of interactions (OH.F and/or OH.N) and molecular symmetry are connected to the hydrogen bond strength and amount of resonance within the hydrogen-bonded system in all circumstances [14, 21].

### Reaction of ELX with B@CQDs validation

The reaction was validated in accordance with ICH and FDA guidelines [28, 29]. Plotting different concentrations of ELX with B@CQDs against RFI was used to investigate the reaction's sensitivity. The calibrated range was found to be 4 – 100 ng mL^−1^ with the regression equation y = 10.0493x + 1585 as shown in Table 1, the lower limit of quantitation (LOQ) was found to be 1.74 ng mL^−1^ and the lower limit of detection (LOD) was determined to be 0.57 ng mL^−1^. The results show that the proposed approach has high sensitivity.

**Table 1:** Analytical parameters for the proposed method for determination of ELX

Parameter	ELX
λ_ex_ (nm)	370
λ_em_(nm)	435
Concentration range (ng mL^−1^)	4–100
Determination coefficient (r^2^)	0.9992
Slope	10.04
Intercept	1585
SD the intercept (Sa)	1.75
LOD (ng mL^−1^)	0.57
LOQ (ng mL^−1^)	1.74

*LOD* Limit of detection,*LOQ* Limit of quantitation

The accuracy of B@CQDs with ELX was investigated using five concentrations (10, 20, 50, 90, and 100 ng mL.^−1^) within the calibration range, the percent of recoveries were ranged from 99.86 to 100.65 and RSD values were ranged from 0.21 to 1.00. The results show that the proposed approach is high accurate. Table 2

**Table 2:** Accuracy and precision results of the proposed method for determination of ELX

Sample number	Taken (ng mL^−1^)	Found (ng mL^−1^)	% Recovery* ± RSD
1	10	10.01	100.10 ± 0.50
2	20.0	20.05	100.25 ± 1.00
3	50.0	50.12	100.24 ± 0.76
4	90.0	89.88	99.86 ± 0.55
5	100.0	100.65	100.65 ± 0.21
Intra-day precision	10	10.10	101.00 ± 0.31
	50	50.06	100.12 ± 0.40
	100	100.22	100.22 ± 0.72
Inter-day precision	10	10.02	100.20 ± 0.82
	50	49.90	99.80 ± 0.33
	100	99.69	99.69 ± 0.80

*Average of three determinations. *RSD* Relative standard deviation

While, the intra-day precision of the presented method was tested at three concentration levels (10, 50 and 100 ng mL^−1^) at three successive measurements. While the inter-day precision was investigated using three concentrations measured as three replicates for three consecutive days. The results obtained refer to excellent repeatability Table 2.

In order to evaluate the interference from plasma, the effect of matrix solution was established with ELX using three levels of quality control samples of the investigated drug. The percent of recovery ± RSD ranged from 94.05 ± 0.99 to 97.90 ± 1.44. The results indicated the absence of interference from the matrix with ELX under different conditions and referred to the high selectivity of the proposed method as shown in Additional file 1: Table S1.

Incurred sample reanalysis (ISR) is a very important parameter to evaluate accuracy and precision of incurred samples in bio-analytical validations using FDA guidelines. In the presented study, the percentage difference between the initial and incurred samples was found to be 3.40%. According to FDA guidelines, the results of incurred samples met the accepted criteria as shown in Additional file 1: Table S2.

### Selectivity of the reaction

The selectivity of the proposed method was studied using external materials as (sucrose, glycine, urea, Ca^2+^, Cu^2+^ and cystine) and different analgesic as tenoxicam, diclofenac. No interference of the external materials was observed as in Additional file 1: Fig S3. It was observed that non-significant enhancement was observed with sucrose, glycine, and urea. However quenching effect was observed with Ca^2+^, Cu^2+^, tenoxicam and diclofenac due to the absence of fluorine atom that forming hydrogen bonding with B@CQDs [14, 21]. The results indicate to the high selectivity of this work.

### Applications of ELX and B@CQDs method

The method was successfully applied in spiked, real human plasma and its formulation. The percent of recovery in spiked human plasma was observed to be 98.80 ± 0.92 as in Table 3.

**Table 3:** Application of the spectrofluorimetric method for determination of ELX in spiked human plasma

Added conc. (ng mL^−1^)	Found (ng mL^−1^)	% Recovery* ± RSD
5	4.89	97.80 ± 0.81
10	9.88	98.80 ± 0.92
20	19.42	97.10 ± 0.84
50	48.08	96.16 ± 1.21
90	88.03	97.82 ± 1.64
100	97.10	97.10 ± 0.79

*Average of six determinations

Determination of ELX in real human plasma (pharmacokinetic study) was carried out about its therapeutic level, peak plasma level (C max) of cited drug was found to be 570 ± 5.32 ng mL^−1^ after administration of ELX 150 mg/ tablet as single oral dose which agrees with other reported one [30]. All the parameters of PK were recorded in Table 4.

**Table 4:** Pharmacokinetic study of ELX using the proposed method

Time (h)	Oral (ng mL^−1^)	Parameters	Results
0.5	320	C_max_ (ng mL^−1^)	570 ± 5.32
1.0	570	T_max_ (h)	1.0 ± 0.10
3.0	500	t _½_ (h)	6.5 ± 1.01
5.0	400	AUC (ng·h mL^−1^)	1290 ± 30.33
6	280		
9	200		
10	100		
15	70		
20	50		
25	42		
30	20		

Besides, B@CQDs was applied for determination of ELX in pharmaceutical dosage form, and the obtained results were found to be satisfactory with a good recovery (99.70 ± 0.69) with t value (1.40) and F value (2.86) compared with other reported method [10]. The evaluation of content uniformity test for ELX was performed by applying the general procedure according to USP guidelines [17, 18]. The content of individual dosage form was analyzed then percentage recoveries were calculated individually. The percent of recovery was recorded at Table 5.

**Table 5:** Content uniformity for ELX (Orilissa® tablets) using the proposed method

Dosage form No	% labeled claim
	Orilissa® tablets (200 mg/tab)
1	99.11
2	100.45
3	98.99
4	99.11
5	100.22
6	98.88
7	100.11
8	99.60
9	99.93
10	100.02
Mean	99.64
SD	0.57
RSD	0.57
Acceptance value (AV)*	1.4
Max. allowed AV (L1)*	15

*Acceptance value = 2.4 × SD

### Comparison of the presented method with the reported methods

Comparing the results in our work with other reported as in Table 6. It was found B@CQDs can serve as a probe for the detection of ELX in a low concentration with higher sensitivity and reliability than other reported methods.

**Table 6:** Comparison reported methods for elagolix with presented method

Method	LOD ng mL^−1^	LOQ ng mL^−1^	Refs.
Fluorimetry	0.57	1.74	Presented study
HPLC	200	500	[9]
Fluorimetry	16.50	50.0	[10]
UPLC-MS/MS	200	500	[11]

## Conclusion

This work presents a sensitive, selective, and low-cost spectrofluorometric method for determination of elagolix (ELX) using B@CQDs with LOQ equal to 1.74 ng mL^−1^. It was successfully applied for determination of ELX in pharmaceutical dosage form, content uniformity and pharmacokinetic study. Pharmacokinetic parameters were established as C_max_ was found to be 570 ± 5.32 ng. mL^−1^ after 1 h, t _½_ was found to be 6.50 h and AUC was found to be 1290 ± 30.33 ng. h. mL^−1^. B@CQDs were confirmed using transmission electron microscopy (TEM), scanning electron microscopy (SEM), X-ray diffraction (PXRD), dynamic light scattering (DLS), and Fourier-transform infrared spectroscopy (FTIR).

## Supplementary Information

**Additional file 1: Fig. S1. **PXRD for undoped carbon quantum dots. **Fig. S2.** optimization of B-CDs reaction **a** effect of pH, **b** effect of volume of buffer, **c** volume of B-CDs using ELX (50 ng mL^-1^), **d** reaction time. **Fig. S3.** Selectivity of B@CQDs to ELX. **Table S1.** Stability and selectivity of ELX in human plasma using different stability conditions. **Table S2.** Incurred sample reanalysis data of ELX.

## Data Availability

All data generated or analyzed during this study are included in this published article [and its additional files].

## Abbreviations

ELX: Elagolix
B@CQDs: Boron doped quantum dots
RFI: Relative fluorescence intensity
C_max_: Maximum plasma concentration
AUC: Area under curve
XPS: X-ray photoelectron spectroscopy
