# Supplementary material for: Ultra-sensitive and selective fluorescence approach for estimation of elagolix in real human plasma and content uniformity using boron-doped carbon quantum dots
Source: BMC Chem. 2022 Aug 4;16(1):58. doi: 10.1186/s13065-022-00849-3 (PMC9351230; doi:10.1186/s13065-022-00849-3)
Supplement: Supplementary file 1 — Additional file 1: Fig. S1. PXRD for undoped carbon quantum dots. Fig. S2. optimization of B-CDs reaction a effect of pH, b effect of volume of buffer, c volume of B-CDs using ELX (50 ng mL-1), d reaction time. Fig. S3. Selectivity of B@CQDs to ELX. Table S1. Stability and selectivity of ELX in human plasma using different stability conditions. Table S2. Incurred sample reanalysis data of ELX. [file 13065_2022_849_MOESM1_ESM.docx]

**Ultra-sensitive and selective fluorescence approach for estimation of elagolix in real human plasma and content uniformity using boron-doped carbon quantum dots**

Baher I. Salman ^a^, Ahmed I. Hassan ^a^, Yasser F. Hassan ^a^, Roshdy E. Saraya ^b^

^a^ Pharmaceutical Analytical Chemistry Department, Faculty of Pharmacy, Al-Azhar University, Assiut branch, Assiut, 71524, Egypt, [**bahersalman@azhar.edu.eg**](mailto:bahersalman@azhar.edu.eg)**,** [**bahersalman2013@yahoo.com**](mailto:bahersalman2013@yahoo.com)

Tel: +201099031345

^b^ Pharmaceutical Analytical Chemistry Department, Faculty of Pharmacy, Port Said University, Port Said 42511, Egypt.

# * Corresponding author: Baher I. Salman

**Email:** [bahersalman@azhar.edu.eg](mailto:bahersalman@azhar.edu.eg)

[bahersalman2013@yahoo.com](mailto:bahersalman2013@yahoo.com)

**Tel.** +201099031345


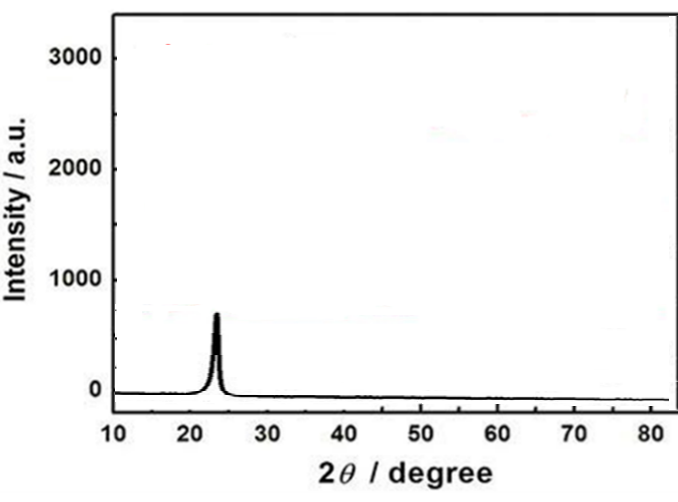
**Fig. S1** PXRD for undoped carbon quantum dots


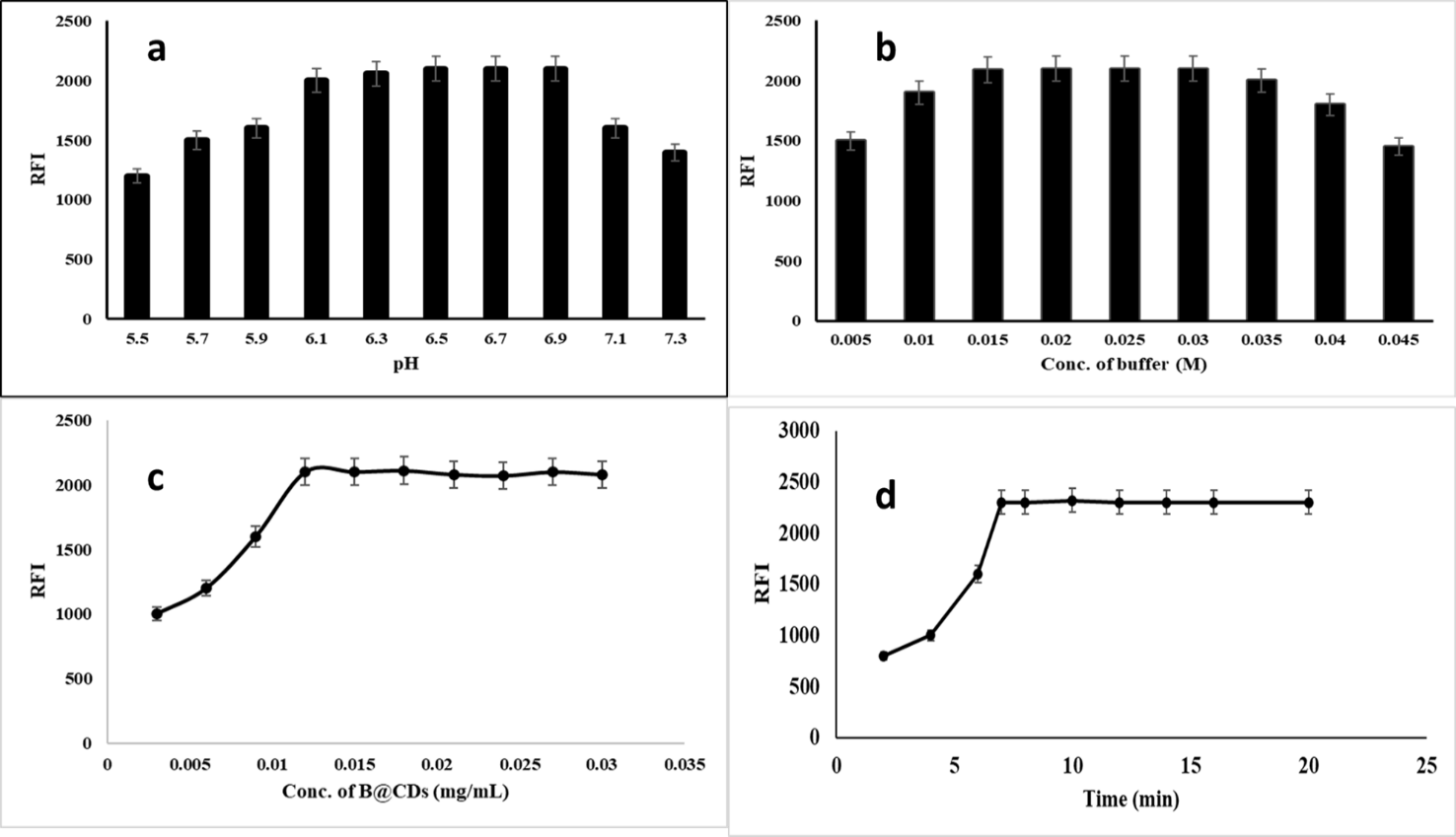


**Fig. S2** optimization of B-CDs reaction a) effect of pH, b) effect of volume of buffer, c) volume of B-CDs using ELX (50 ng mL^-1^), d) reaction time.


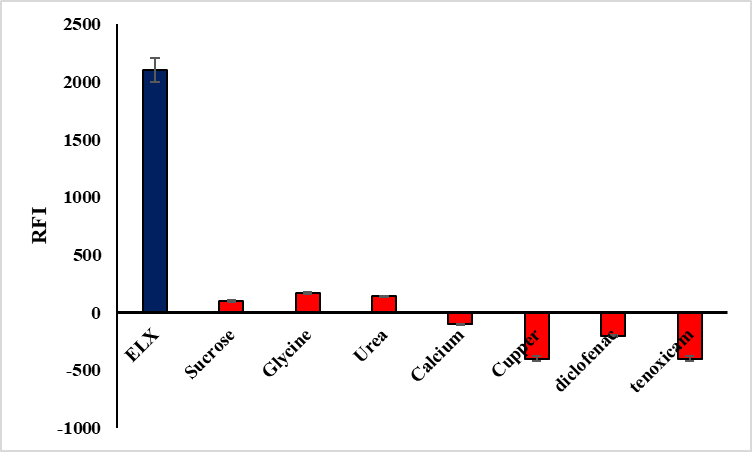


**Fig. S3** Selectivity of B@CQDs to ELX

**Table S1** Stability and selectivity of ELX in human plasma using different stability conditions.

| **Conditions** | **% Recovery ± RSD (n = 5)** | | |
| --- | --- | --- | --- |
| **Concentrations** | **LQC**  **10 ng mL^-1^** | **MQC**  **50 ng mL^-1^** | **HQC**  **90 ng mL^-1^** |
| Three Freeze–thaw cycle stability (-24°C) | 94.39 ± 1.33 | 96.11 ± 1.40 | 94.99 ± 1.14 |
| Long-term stability (1 month at -24°C) | 95.22 ± 1.90 | 95.43 ± 1.30 | 95.30 ± 1.82 |
| Short-term stability (12 hr at -24°C) | 96.90 ± 1.00 | 96.09 ± 1.71 | 96.55 ± 1.67 |
| Post-preparative stability (6 hr at room temperature 25 °C) | 94.05 ± 0.99 | 97.90 ± 1.44 | 96.21 ± 1.72 |
| Post-preparative stability (12 hr at room temperature 25 °C) | 96.50 ± 1.62 | 95.55 ± 1.49 | 96.60 ± 1.05 |

**Table S2** Incurred sample reanalysis data of ELX.

| **Sample** | **Initial concentration**  **% Recovery* ± SD** | **Incurred concentration**  **% Recovery* ± SD** | **% Deviation** |
| --- | --- | --- | --- |
| **1** | 97.31 ± 1.60 | 94.00 ± 2.10 | - 3.40 |
| **2** | 96.35 ± 2.11 | 95.11 ± 2.07 | - 1.28 |
| **3** | 95.02 ± 2.04 | 94.23 ± 1.76 | - 0.83 |

**^*^:** Mean of six determinations.
